# Intranasal Administration of SARS-CoV-2 ORF8 Accessory Protein Increases Blood Pressure and Oxidative Stress in Different Tissues of Male BALB/c Mice

**DOI:** 10.3390/v18040440

**Published:** 2026-04-05

**Authors:** Karla A. Pavon-Martinez, Giovani Visoso-Carvajal, Rebeca Campi-Caballero, Jazmín García-Machorro, Judith Espinosa-Raya

**Affiliations:** 1 Laboratorio Multidisciplinario en Ciencias Biomédicas, Escuela Superior de Medicina, Instituto Politécnico Nacional, Plan de San Luis y Salvador Díaz Mirón s/n, Casco de Santo Tomás, Ciudad de México C.P. 11340, Mexico; karlapavon_15@hotmail.com (K.A.P.-M.); beckycampi@yahoo.com.mx (R.C.-C.); 2 Laboratorio de Medicina de la Conservación, Escuela Superior de Medicina del Instituto Politécnico Nacional, Plan de San Luis y Salvador Díaz Mirón s/n, Casco de Santo Tomás, Ciudad de México C.P. 11340, Mexico; carvajalgv@gmail.com

**Keywords:** SARS-CoV-2, ORF8, oxidative stress, COVID-19, arterial blood pressure

## Abstract

SARS-CoV-2 is the etiological agent responsible for COVID-19. While most research has focused on structural proteins, the accessory protein Open Reading Frame 8 (ORF8) has attracted attention for its role in immune evasion and the induction of a cytokine storm. Although the exact mechanisms underlying viral pathogenicity remain to be elucidated, oxidative stress has been proposed as a key contributing factor. In this study, we evaluated the effect of intranasal administration of ORF8 on arterial blood pressure and the antioxidant system in different organs of male BALB/c mice at 2- or 8 weeks post-administration. A significant increase in blood pressure and renal total antioxidant capacity was observed in the 8-week group, and decreased catalase activity in the prefrontal cortex was observed in the 2-week group. These findings suggest that ORF8 may contribute to long-term renal alterations and potentially to mechanism relevant to cognitive dysfunction associated with COVID-19.

## 1. Introduction

Severe acute respiratory syndrome coronavirus 2 (SARS-CoV-2) was identified as the etiological agent of Coronavirus Disease 2019 (COVID-19) in late December 2019, leading the World Health Organization (WHO) to declare it a pandemic in March 2020 due to its genetic sequence indicating a novel coronavirus, its ability to cross the species barrier, and its efficiency in spreading from human to human [1]. As of 15 February 2026, over 779,142,314 cases and 7,111,892 deaths have been reported worldwide, with infection rates increasing during winter month [2]. While many individuals experience flu-like symptoms, some remain entirely asymptomatic. Those who experience the worst outcomes often have underlying comorbidities or are immunosuppressed. In addition to the acute infection, some patients report persistent symptoms lasting more than three months, up to a year after recovery from the acute illness, a condition known as long COVID [1,3,4].

Most research has focused on the whole virus or its structural and non-structural proteins, often overlooking accessory proteins [5]. Although accessory proteins have little to no relevance for in vitro replication, they play critical roles in regulating proliferation, pathogenicity, and immune evasion in vivo [3,6]. Among SARS-CoV-2 accessory proteins, ORF8 has attracted attention due to its low homology with proteins from earlier coronaviruses. ORF8 consists of 121 amino acids, including an N-terminal transmembrane domain for endoplasmic reticulum (ER) translocation and a nuclear region with eight antiparallel β-sheets and an immunoglobulin-like domain. Three disulfide bonds stabilize this protein and usually form homodimers. This structure promotes ER stress and a prooxidant environment, leading to the buildup that triggers the unfolded protein response (UPR) and raises chaperone levels to restore ER homeostasis. Another important feature of ORF8 is immune evasion; it can bind to the major histocompatibility complex I (MHC-I) while residing in the ER, which leads to its degradation [7]; or because it can antagonize interferon type I (IFN-I), avoiding nuclear translocation of interferon regulatory factor 3 (IRF3) [8]. Moreover, evidence indicates that ORF8 facilitates a cytokine storm by activating NLRP3 and interacting with IL17RA, thereby elevating the production of proinflammatory cytokines [9,10,11].

Oxidative stress (OS) plays a key role in the progression of COVID-19 and its systemic effects. It is a pathological state resulting from an imbalance between reactive oxygen species (ROS) and antioxidant defense mechanisms [12]. In mitochondria, ROS are endogenously generated by the electron transport chain, in which electrons are transferred to oxygen, producing superoxide anions. These anions can be converted to hydrogen peroxide and hydroxide, which are then eventually converted into water and oxygen by enzymes, if not transformed, hydrogen peroxide could undergo the Fenton reaction that leads to hydroxyl radical. Since ROS act as prooxidants, they can interact with and oxidize macromolecules like proteins, DNA, and lipids. Enzymes such as oxidases and peroxidases, which can be triggered by diet or infections, further boost ROS levels. The antioxidant system consists of both enzymatic and non-enzymatic components, with the latter comprising small molecules that trap ROS and prevent macromolecular oxidation [13,14].

On the other hand, SARS-CoV-2 has been shown to elevate blood pressure during both the acute phase and long COVID-19. This effect is primarily mediated by binding to Angiotensin-converting enzyme 2 receptor (ACE2), a functional protein anchored to the cell surface. It acts as an essential enzyme for the renin–angiotensin–aldosterone system (RAAS), which regulates systemic blood pressure. The binding of Angiotensin II (Ang II, a hormone and key component of RAAS) to the Angiotensin II Type 1 Receptor (AT1R) not only increases OS but also induces vasoconstriction and peripheral vascular resistance, thereby elevating blood pressure and altering cardiac and renal function [15,16]. Collectively, heightened OS and increased vascular peripheral resistance can contribute to the development of multiple organ dysfunction syndrome (MODS), as observed in both acute COVID-19 and long-term cases [17].

Animal models help isolate the effects of individual viral proteins and clarify their contributions to clinical outcomes in humans. For this reason, this study aims to investigate the effect of intranasal administration of SARS-CoV-2 ORF8 accessory protein on the antioxidant system activity in the prefrontal cortex, lungs, heart, liver, and kidneys of male BALB/c mice, as well as its association with the development of cardiovascular complications. These tissues are frequently implicated in both acute and long COVID-related pathology.

## 2. Materials and Methods

### 2.1. Reagents

The recombinant SARS-CoV-2 ORF8 accessory protein, was obtained from VIROGEN (Boston, MA, USA, Cat. 00230 V), is expressed in *Escherichia coli*, and the product is supplied at a concentration of 1 mg/mL in PBS. The commercial supplier certifies endotoxin levels < 0.1 EU/μg.

Commercially available kits were used to determine total antioxidant capacity (TAC) (Invitrogen, Frederick, MD, USA, Cat. EIAFECL2), superoxide dismutase (SOD) (Invitrogen, Frederick, MD, USA, Cat. EIASODC) and catalase (CAT) (Invitrogen, Frederick, MD, USA, Cat. EIACATC) activities, as well as total glutathione concentration (GSHT) (Cayman Chemical, Ann Arbor, MI, USA, Cat. 703002) and malondialdehyde quantification using TBARS kit (Cayman Chemical, Saint Louis, MI, USA, Cat. 10009055).

### 2.2. Animals

Twenty-four male BALB/c mice (8–10-week-old) were provided from the Office of Animal Resource Facility of Universidad Autónoma del Estado de Hidalgo. The mice were housed in a standard environment (temperature: 22 ± 1 °C; relative humidity: 55 ± 5%) in the SuperMouse 750TM Micro-isolator (Plexx B.V. Güeldres, The Netherlands), with ad libitum access to food and water in accordance with the Official Mexican Standard NOM-062-ZOO-1999. for laboratory animal care. After a week of acclimatization, mice underwent intranasal administration using the procedure described by Hanson and collaborators [18] for a week. The experimental protocol (ESM-CBS-01/05-011-2025) was approved by the Institutional Biosafety Committee. Measures were taken to reduce animal numbers and discomfort. All procedures were conducted between 10:00 and 15:00, with independent groups for each experiment.

### 2.3. Intranasal Administration of SARS-CoV-2 ORF8 Accessory Protein

Mice were randomly assigned to four groups (n = 6 each): 2 groups for 2 weeks (short-term) and 2 groups for 8 weeks (long-term). Each period included a control group inoculated with PBS and another with ORF8 accessory protein. Each mouse received an intranasal dose of 25 μg in a total volume of 25 μL to simulate the entry route of the virus into the body. Three inoculations were carried out, with a 1-week interval between them. All animals were immobilized with the non-dominant hand and positioned with the neck parallel to the floor to perform intranasal administration using a pipette with the dominant hand, as described by Hanson and collaborators [18]. Blood samples were collected from the tail at time 0 (before inoculation with ORF8, pre-immune) and at day 7 after last inoculation (hyperimmune), and the serum was stored at −20 °C.

### 2.4. Detection of Antibodies Against ORF8 Protein

The antibodies against ORF8 protein were measured by ELISA in 96-well polyvinyl plates (Costar) coated overnight with ORF8 protein (2 μg/mL). Then, 1:25 dilutions of pre-immune and hyperimmune sera were incubated overnight at 4 °C in duplicate. After incubation with 1:5000 goat anti-mouse immunoglobulin peroxidase-conjugated secondary antibody anti-IgG (Invitrogen, Waltham, MA, USA), H_2_O_2_, and 2,2′-azino-bis (3-thylbenzothiazoline-6-sulfonic acid) (ABTS) (Sigma-Aldrich, St. Louis, MO, USA) were added as substrates. The absorbance values were determined at 405 nm.

### 2.5. Arterial Blood Pressure Measurement

Systolic blood pressure (SBP), diastolic blood pressure (DBP), and mean blood pressure (MBP) were assessed using a computerized tail-cuff method (CODA 4, Kent Scientific Corporation, Torrington, CT, USA) at the end of 2- or 8 weeks post-administration. The mice were trained for two consecutive days by placing them in a plastic rack mounted on a thermostatically controlled hot plate maintained at 35 °C. The habituation period was followed by a test session on the warming platform at level 2 (around 37 °C), during which the mice were placed in the holders, and the occlusion cuff and VPR cuff were adjusted as described by Daugherty and collaborators [19]. The software parameters were 5 cycles of acclimatation, 1 set, 15 cycles per set, and a deflation time of 20 s. All blood pressure measurements were made between 10:00 h and 14:00 h.

### 2.6. Tissue Preparation and Protein Quantification

A day after blood pressure measurement, mice were euthanized by decapitation with a guillotine, and their prefrontal cortex, lungs, heart, liver, and kidneys were quickly dissected and stored in an ultra-low temperature freezer at −80 °C. When needed, samples were collected until later use.

The tissues were thawed and subsequently lysed using an electric homogenizer and lysis buffer (Sigma-Aldrich: Tris-HCl 10 mM, Cat. T1503; triton X100 1%, Cat. 1.086603; sodium azide 0.02%, Cat. S8032; EDTA 1 mM, Cat. E9884), centrifuged at 12,000 rpm, at 4 °C for 5 min. The supernatant was separated, and protein was quantified by the Bradford method, using the Protein Assay Dye Reagent Concentrate (Bio-Rad, Hercules, CA, USA, Cat. 0469311001) with bovine serum albumin as a standard. The results were read using the iMark™ Microplate Absorbance Reader (Bio-Rad, Cat. 5000006). The supernatants were stored −70 °C with the cOmplete™ Protease Inhibitor Cocktail (Sigma, Cat. 18-1130), until colorimetric determination.

### 2.7. Measurements of Total Antioxidant Capacity (TAC), Superoxide Dismutase (SOD) and, Catalase (CAT) Activity, and Total Glutathione Concentration

Commercially available kits were used to determine total antioxidant capacity (TAC) (Invitrogen, Cat. EIAFECL2), superoxide dismutase (SOD) (Invitrogen, Cat. EIASODC) and catalase (CAT) (Invitrogen, Cat. EIACATC) activities, as well as total glutathione concentration (GSHT) (Cayman Chemical, Cat. 703002). The manufacturer’s instructions were strictly followed during every assay, and results were read using the iMark™ Microplate Absorbance Reader (Bio-Rad, Cat. 5000006).

The TAC results are reported as equivalent to ferrous chloride (FeCl2 µM). SOD and CAT enzymatic activities are reported as U/mL (units per milliliter) of each enzyme. GSHT is reported as equivalent to glutathione (GSH µM). All quantifications were adjusted to mg of protein in each determination.

### 2.8. Quantification of Lipid Peroxidation

Malondialdehyde (MDA) is one of the end products of polyunsaturated fatty acid peroxidation and is considered a marker of oxidative stress. MDA was measured in kidney and cerebral cortex supernatant using the assay kit (cat. no. 10009055, Cayman Chemical). Based on the reaction of thiobarbituric acid reactive substances (TBARS) and MDA to form the MDA-TBA adduct, the manufacturer’s instructions were followed. After the reaction was complete, the absorbance was measured at 540 nm [20]. The MDA concentration was calculated according to the standard curve, and the results were expressed in µM.

### 2.9. Statistical Analyses

All statistical analyses were performed using Sigmaplot software (version 14.0, Systat Software, Inc., San Jose, CA, USA). The data are expressed as the means  ±  SEM, and a *p*-value < 0.05 was considered statistically significant. Data normality was evaluated using the Shapiro–Wilk test. The results of IgG antibodies against ORF8 were analyzed by paired *t* test. The results of blood pressure, TAC, SOD, CAT, GSHT and MDA were analyzed using a two-way analysis of variance (ANOVA) with ORF8 and TIME as independent variables, followed by Bonferroni post hoc multiple-comparison tests.

## 3. Results

### 3.1. Detection of Antibodies Against the ORF8 Protein

As an indirect measure of the temporary presence of the ORF8 protein (inoculated via intranasal route) in the mouse organism, the serum IgG antibody response was determined. ELISA plates were coated with recombinant ORF8 protein, and the total IgG antibody response was measured in the mice’s serum. Statistical analysis was performed using a paired *t*-test (preimmune sera vs. hyperimmune sera). The results show a significant difference (*p* ˂ 0.0001) before and after inoculation. See results in Supplementary Figure S1.

### 3.2. Blood Pressure Measurement

**Figure 1:**
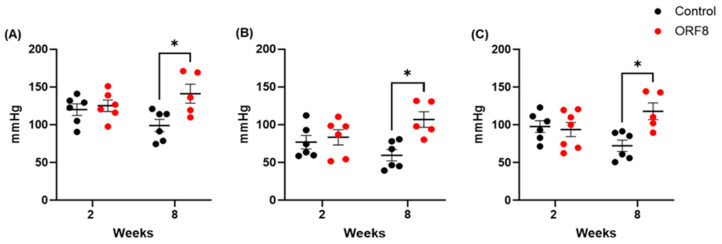
Systolic blood pressure (**A**), diastolic blood pressure (**B**), and mean blood pressure (**C**) of BALB/c mice; 2 and 8 weeks after administration of SARS-CoV-2 ORF8 accessory protein. All data are mean ± SEM; n = 6. * *p* < 0.05 by two-way ANOVA followed by Bonferroni post hoc tests.

To evaluate the impact of intranasal administration of ORF8 protein on the cardiovascular system, arterial blood pressure was measured. The results are shown in Figure 1. The SBP data analysis showed a significant main effect of ORF8 (F = 6.98, *p* < 0.05). However, there was no main effect of TIME (F = 0.08, *p* = 0.77) or ORF8 × TIME interaction (F = 4.29, *p* = 0.05). For DBP and MBP, the data showed a significant main effect of ORF8 (F = 8.60, *p* < 0.01 and F = 5.36, *p* < 0.03, respectively) and in the ORF8 × TIME interaction (F = 4.9, *p* < 0.05 and F = 7.57, *p* < 0.05, respectively). There was no effect of TIME (F = 0.12, *p* = 0.73 and F = 0.005, *p* = 0.94, respectively). In general, the post hoc test showed that ORF8 increased SBP (*p* < 0.05), DBP (*p* < 0.05), and MBP (*p* < 0.05) in the long-term groups.

### 3.3. Effects of SARS-CoV-2 ORF8 Accessory Protein on Total Antioxidant Capacity (TAC), Superoxide Dismutase (SOD), Catalase (CAT), and Total Glutathione Concentration

#### 3.3.1. Total Antioxidant Capacity

**Figure 2:**
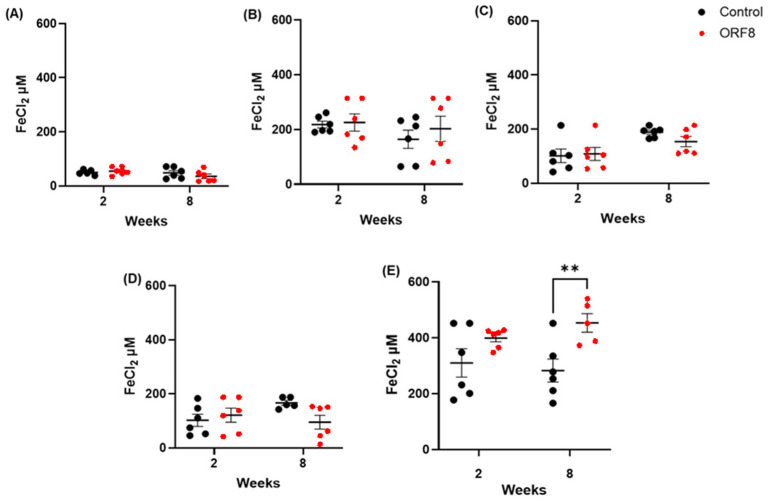
Total antioxidant capacity (TAC) in prefrontal cortex (**A**), lungs (**B**), heart (**C**), liver (**D**), and kidneys (**E**) of BALB/c mice 2 and 8 weeks after administration of SARS-CoV-2 ORF8 accessory protein. All data are mean ± SEM; n = 6. ** *p* < 0.01 by two-way ANOVA followed by Bonferroni post hoc tests.

The results of the TAC, expressed in FeCl_2_ equivalents in μM, are presented in Figure 2. No significant difference was found between the treatment groups in the prefrontal cortex (ORF8: F = 0.31, *p* = 0.58; TIME: F = 2.02, *p* = 0.17; ORF8 × TIME: F = 1.45, *p* = 0.24), lung (ORF8: F = 0.49, *p* = 0.49; TIME: F = 1.35, *p* = 0.26; ORF8 × TIME: F = 0.22, *p* = 0.65), and liver (ORF8: F = 1.33, *p* = 0.26; TIME: F = 0.72, *p* = 0.40; ORF8 × TIME: F = 3.97, *p* = 0.06). In the heart, data analysis showed a main effect of TIME (F = 10.85, *p* < 0.01) but not effect in ORF8 (F = 0.47, *p* = 0.50) nor in the ORF8 × TIME interaction (F = 1.07; *p* = 0.31); the Bonferroni tests did not reveal any relevant differences. In the kidney, there was a significant main effect of ORF8 (F = 11.74, *p* < 0.01) but not in TIME (F = 0.12, *p* = 0.73) or ORF8 × TIME interaction (F = 1.16, *p* = 0.29); the Bonferroni test showed a higher antioxidant capacity in the long-term ORF8-treated group compared with long-term control.

#### 3.3.2. Superoxide Dismutase Activity

**Figure 3:**
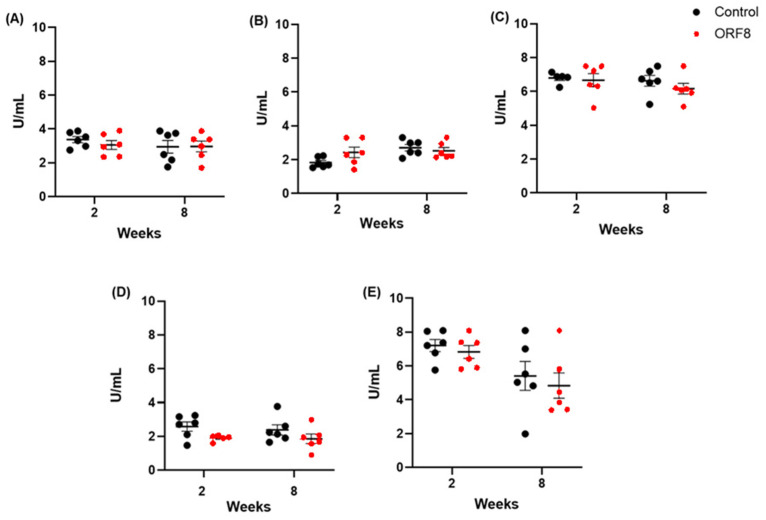
Superoxide dismutase activity (SOD) in prefrontal cortex (**A**), lungs (**B**), heart (**C**), liver (**D**), and kidneys (**E**) of BALB/c mice 2 and 8 weeks after administration of SARS-CoV-2 ORF8 accessory protein. All data are mean ± SEM; n = 6. *p* > 0.05 by two-way ANOVA.

Figure 3 shows the results of the quantification of SOD enzymatic activity, expressed in U/mL. No significant difference was found in the prefrontal cortex (ORF8: F = 0.26, *p* = 0.6; TIME: F = 0.76, *p* = 0.57; ORF8 × TIME: F = 0.34, *p* = 0.57) and heart (ORF8: F = 0.90, *p* = 0.35; TIME: F = 1.08, *p* = 0.31; ORF8 × TIME: F = 0.28, *p* = 0.60). In the liver, there was a significant main effect of ORF8 (F = 5.2, *p* < 0.05), but no effect of TIME (F = 0.21, *p* = 0.65) or ORF8 × TIME interaction (F = 0.08, *p* = 0.78). In the lung, there was a significant main effect of TIME (F = 5.05, *p* < 0.05), but not effect of ORF8 (F = 0.95, *p* = 0.34) or ORF8 × TIME interaction (F = 3.30, *p* = 0.08. In the kidney, a significant main effect was observed for TIME (F = 0.02, *p* < 0.01), but no effect of ORF8 (F = 0.95, *p* = 0.34) or ORF8 × TIME interaction (F = 3.30, *p* = 0.88). The Bonferroni tests did not reveal any relevant differences between the groups.

#### 3.3.3. Catalase Activity

**Figure 4:**
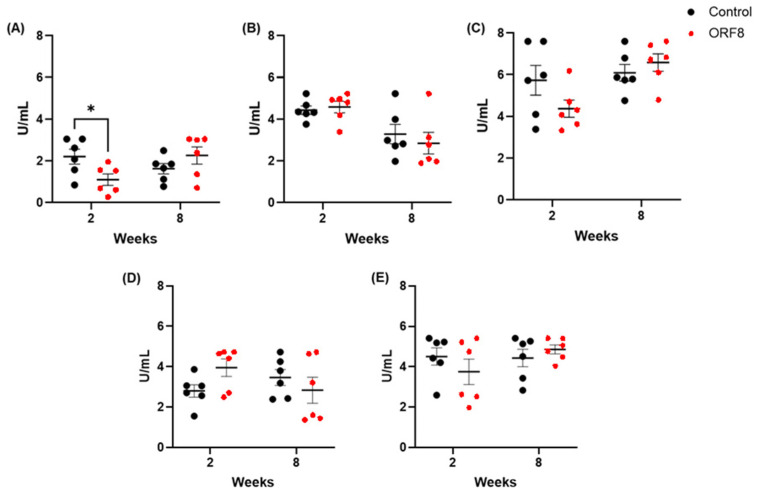
Catalase activity (CAT) in prefrontal cortex (**A**), lungs (**B**), heart (**C**), liver (**D**), and kidneys (**E**) of BALB/c mice 2 and 8 weeks after administration of SARS-CoV-2 ORF8 accessory protein. All data are mean ± SEM; n = 6. * *p* < 0.05 by two-way ANOVA followed by Bonferroni post hoc tests.

The results of the enzymatic activity of CAT are presented in Figure 4. No significant difference was found in the liver (ORF8: F = 3.68, *p* = 0.09; TIME: F = 0.48, *p* = 0.51; ORF8 × TIME: F = 0.02, *p* = 0.89) and kidney (ORF8: F = 0.13, *p* = 0.71; TIME: F = 1.31, *p* = 0.27; ORF8 × TIME: F = 1.71, *p* = 0.21). In the prefrontal cortex was found a significant ORF8 × TIME interaction (F = 7.00, *p* < 0.05) but not effect of ORF8 (F = 0.50, *p* = 0.49) nor TIME (F = 0.77, *p* = 0.39); the post hoc test showed a decreased activity in the short-term ORF8-treated group compared with short-term control (*p* < 0.05)). In the lung, there was a significant main effect of TIME (F = 13.81, *p* < 0.01) but not effect of ORF8 (F = 0.14, *p* = 0.71) or ORF8 × TIME interaction (F = 0.58, *p* = 0.45); the post hoc test did not reveal any relevant differences. In the heart, there was a significant main effect of TIME (F = 6.57, *p* < 0.05) but no effect of ORF8 (F = 0.75, *p* = 0.40) or ORF8 × TIME interaction (F = 3.38, *p* = 0.08); the Bonferroni tests did not reveal any relevant differences between the groups.

#### 3.3.4. Total Glutathione Concentration

**Figure 5:**
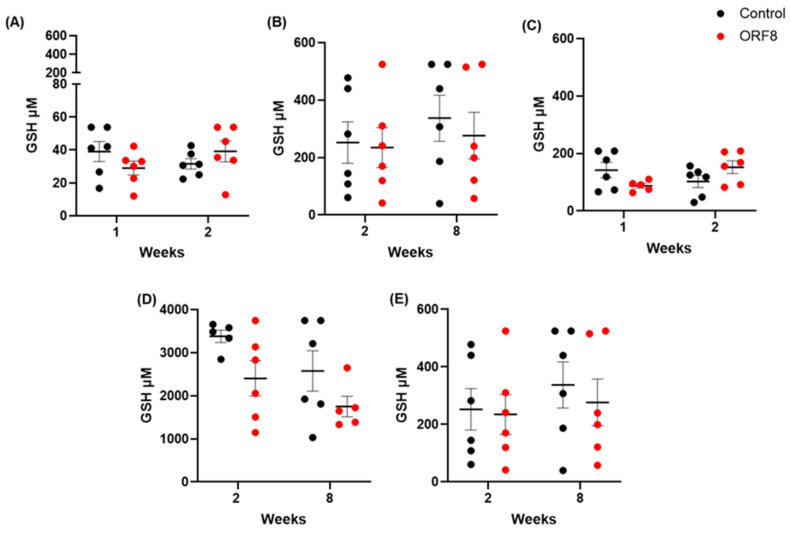
Total glutathione concentration in prefrontal cortex (**A**), lungs (**B**), heart (**C**), liver (**D**), and kidneys (**E**) of BALB/c mice 2 and 8 weeks after administration of SARS-CoV-2 ORF8 accessory protein. All data are mean ± SEM; n = 6. *p* > 0.05 by two-way ANOVA.

The results of the determination of the total glutathione concentration are presented in Figure 5. No significant difference was found in the prefrontal cortex (ORF8: F = 0.05, *p* = 0.81; TIME: F = 0.07, *p* = 0.78; ORF8 × TIME: F = 2.97, *p* = 0.10), lung (ORF8: F = 0.27, *p* = 0.61; TIME: F = 0.69, *p* = 0.42; ORF8 × TIME: F = 0.08, *p* = 0.78) and kidney (ORF8: F = 0.27, *p* = 0.61; TIME: F = 0.69, *p* = 0.41; ORF8 × TIME: F = 0.08, *p* = 0.78). In the heart, there was a significant ORF8 × TIME interaction (F = 5.96, *p* < 0.05), but no main effect of ORF8 (F = 0.01, *p* = 0.91) and TIME (F = 0.33, *p* = 0.57); the post hoc Bonferroni tests did not reveal any significant group differences. In the liver, there was a significant main effect of ORF8 (F = 6.10, *p* < 0.05) but no main effect of TIME (F = 3.97, *p* = 0.06) nor in the ORF8 × TIME interaction (F = 0.04, *p* = 0.84); the post hoc Bonferroni tests did not reveal any significant group differences.

### 3.4. Effect of SARS-CoV-2 ORF8 Accessory Protein on Malondialdehyde (MDA)

**Figure 6:**
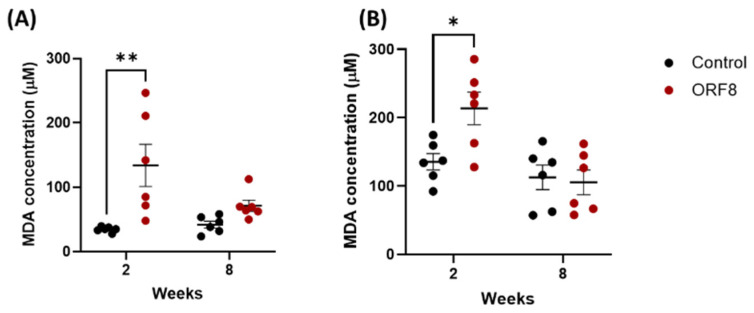
Malondialdehyde concentration in prefrontal cortex (**A**), and kidneys (**B**) of BALB/c mice 2 and 8 weeks after administration of SARS-CoV-2 ORF8 accessory protein. All data are mean ± SEM; n = 6. ** *p* < 0.003 and * *p* < 0.03 by two-way ANOVA.

The results of the determination of the MDA concentration are presented µM in Figure 6. Significant difference was found in the prefrontal cortex in main effect of ORF8 (F = 13.96, *p* = 0.001), but no in TIME (F = 2.58, *p* = 0.12) and ORF8 × TIME interaction (F = 4.12, *p* = 0.05). In the kidney, there was a significant difference when comparing factor TIME (F = 12.58, *p* = 0.002) or ORF8 × TIME interaction (F = 5.35, *p* = 0.03) but not in main effect of ORF8 (F = 3.67, *p* = 0.06). In both organs (prefrontal cortex and kidney) a significant difference was found (*p* = 0.003 and *p* = 0.03, respectively) when the means were compared at two weeks in the control group vs. ORF8.

## 4. Discussion

SARS-CoV-2, the etiological agent of COVID-19, predominantly causes respiratory symptoms due to its mode of entry; however, evidence indicates it can also cause systemic organ damage. This phenomenon is associated not only with the distribution of ACE2 receptors but also with the capacity of the virus to replicate and infect adjacent cells across various tissues. Such effects have been chiefly documented in lung, gastrointestinal, hepatic, renal, and neuronal cells [21]. Since these findings are primarily derived from in vitro studies and histopathological damage has been observed across multiple organs, this study employed an in vivo model to investigate the contribution of the ORF8 protein to oxidative stress in different tissues.

Intranasal inoculation was chosen to simulate SARS-CoV-2 natural entry. Unlike methodologies employed in other investigations, anesthesia was omitted to eliminate potential confounding variables, particularly those affecting central nervous system studies [22,23]. Two post-exposure time points (2 and 8 weeks) were selected to evaluate changes relative to control groups during both the acute phase and a period mimicking long COVID. This design was informed by the cognitive analyses of Fontes-Dantas et al. [24], who distinguished between acute (lasting up to two weeks) and long-term post-infection effects (beginning a month after recovery). Selecting these intervals allowed for observation of both transient and potentially persistent modifications due to ORF8 exposure, analogous to timepoints used in other behavioral and inflammatory studies [25].

SARS-CoV-2 is known to increase blood pressure during acute infection, mainly through its interaction with ACE2 receptor, which dysregulates the RAAS, increasing Ang II binding to its receptor and promoting the production of inflammatory cytokines [26,27]. This cascade can contribute to cardiovascular risk, as evidenced in intensive care unit patients and in those with long-term effects [28,29]. Although direct ACE2–ORF8 interaction has not been reported, ORF8-induced cytokines may promote thrombosis through endothelial inflammation, and elevated cytokines can also enhance ROS production [30,31,32,33]. Systolic pressure rise after infection could result from cytokine induction or ER stress in heart and endothelial cells [10,28,29,30,31,34]. Diastolic pressure reflects vessel elasticity and may worsen due to oxidation of endothelial nitric oxide—driven by increased ROS—which damages vessels, leading to chronic endotheliopathy and higher peripheral resistance [15,35,36]. Elevated mean arterial pressure suggests increased organ perfusion requirements, possibly indicating multisystemic damage [30,37].

Regarding the TAC, our data showed that the ORF8 protein significantly increased renal TAC over the long term. While organ-specific changes have not been documented in patients, serum TAC has been shown to decrease in individuals with long COVID, particularly those with increased lipid oxidation products [38]. This phenomenon may be attributable to transient ROS production that initially remains within the limits of antioxidant defenses but eventually exceeds those defenses, potentially due to protein-induced inflammation or direct ROS generation by the protein itself. Although comparable findings have not been previously reported, our results may relate to the development of acute kidney injury (AKI) observed during and following COVID-19 infection [39]. Similarly, our data align with studies in experimental animals, which have demonstrated increases in markers of tubular damage, renal injury, fibrosis, and proinflammatory cytokines [40,41]. Collectively, these findings suggest that excessive ROS can induce renal damage and that elevated TAC represents a compensatory regulatory response.

Reduced SOD activity has been documented in individuals with COVID-19, correlating with both disease severity and markers of cardiac and hepatic injury [42]. Nevertheless, our results indicate that the ORF8 protein is not a contributing factor to these changes; rather, the variations observed are attributable to the age of the mice studied [43].

The CAT activity results show that ORF8 administration decreases its activity in the prefrontal cortex in the short term. This finding is consistent with what was reported by Liu et al. [40], who administered the SARS-CoV-2 virus to transgenic K18-hACE2 C57BL/6J mice that were sacrificed after 5 days and found a higher viral load in the prefrontal cortex than in the lungs, as well as an increase in protein expression disorders and electron transport chain activity. Although their results do not mention CAT activity, they report an increase in electron transport chain activity, which could increase superoxide anion production.

In the quantification of total glutathione, no significant difference was found in any of the tissues. Although it has been shown that the virus can cause a decrease in both glutathione and the dipeptide ɣ-glutamylcysteine [44], and that in patients in the acute phase of COVID-19 it is decreased, but not in those with long COVID, it can be suggested that the ORF8 protein does not alter this molecule of the non-enzymatic system. Given the increase in TAC in the kidneys, this protein could alter other non-enzymatic molecules, such as uric acid, which is one of the most important contributors to TAC [39].

We found changes in antioxidant capacity and catalase activity in kidney and prefrontal cortex tissues, respectively. However, changes in antioxidant enzyme activity do not necessarily equate to oxidative stress. Therefore, MDA was measured as a marker of oxidative stress. In both tissues, we found an increase in MDA two weeks post-inoculation with ORF8. Furthermore, an increase in oxidative stress has been detected in acute viral infection [45]. This suggests that the ORF8 protein is one of the viral antigens involved in inducing oxidative stress as a short-term (2-weeks) damage mechanism [46]. After 8 weeks post-inoculation, no increase in MDA was found, indicating that the body compensates for the damage caused by the ORF8 protein, or it may even be because exposure to this protein was discontinued after three inoculations.

Finally, it is important to mention that the ORF8 protein used was expressed in a prokaryotic system; it is not the complete virus with deletions, or a protein expressed in a eukaryotic system, as in other experiments involving it, so the results presented here may be diminished, since glycosylation in Asn78 would be lacking, for example. But it can also be secreted without glycosylation, which generates an inflammatory response by binding to the IL17R receptor, triggering signaling and cytokine release [10,11]. This is a possible explanation for why the changes in the redox system in some organs are not noticeable, and why the mechanism might be more inflammatory than oxidative.

In summary, the results of this study suggest that ORF8 administration can alter the redox system, potentially contributing to long-term renal changes and mechanisms relevant to cognitive dysfunction associated with COVID-19. However, a limitation of this study is that it does not provide direct biodistribution measurements of ORF8. Nevertheless, IgG antibodies against the ORF8 protein were detected in serum, indicating that the protein did indeed contact with the mucosa and was recognized and presented in an MHC-II context [47].

Another limitation is that ROS production was not directly measured. However, MDA was measured as a marker of oxidative stress and the effect caused by the peroxidation of polyunsaturated fatty acids [48].

## 5. Conclusions

Overall, our results suggest that the ORF8 administration is associated with alterations in antioxidant parameters in vivo. In addition, it can be suggested that two organs may be sensitive to oxidative damage induced by this protein: the kidney and the central nervous system. In turn, the increase in kidney oxidative stress could be related to elevated systemic blood pressure. However, as no similar report is found in the literature, this finding warrants further investigation.

## Data Availability

The original data presented in the study are openly available at the following address: https://drive.google.com/drive/u/0/folders/15CTVFPQMAn61JsnzlCIT2bo6L7QMKZVn (accessed on 2 April 2026).

## Supplementary Materials

The following supporting information can be downloaded at: https://www.mdpi.com/article/10.3390/v18040440/s1, Figure S1: IgG antibodies against the ORF8.

## Abbreviations

The following abbreviations are used in this manuscript:

ACE2	Angiotensin-converting enzyme 2 receptor ACE2
AKI	Acute kidney injury
Ang II	Angiotensin II
AT1R	Angiotensin II Type 1 Receptor
COVID-19	Coronavirus Disease 2019
DBP	Diastolic blood pressure
ER	Endoplasmic reticulum
IL17RA	Interleukin 17 receptor A
IFN-I	Interferon type I
IRF3	Interferon regulatory factor 3
MBP	Mean blood pressure
MDA	Malondialdehyde
MHC-I	Major histocompatibility complex I
MHC-II	Major histocompatibility complex II
MODS	Multiple organ dysfunction syndrome
ORF8	Open Reading Frame 8
OS	Oxidative stress
RAAS	Renin–angiotensin–aldosterone system
ROS	Reactive oxygen species
SARS-CoV-2	Severe acute respiratory syndrome coronavirus 2
SBP	Systolic blood pressure
TBARS	Thiobarbituric acid reactive substances
UPR	Unfolded protein response
WHO	World Health Organization
