# Supplementary material for: Intranasal Administration of SARS-CoV-2 ORF8 Accessory Protein Increases Blood Pressure and Oxidative Stress in Different Tissues of Male BALB/c Mice
Source: Viruses. 2026 Apr 5;18(4):440. doi: 10.3390/v18040440 (PMC13120315; doi:10.3390/v18040440)
Supplement: Supplementary file 1 [file viruses-18-00440-s001.zip › viruses-4156360-supplementary.pdf]

To demonstrate that the ORF8 protein had been inoculated and in contact with the mice, IgG antibodies were determined in sera. The results are shown in Supplementary Figure S1. Optical density is directly proportional to the presence of antibodies, and paired sera from mice were compared before immunization with ORF8 (pre-immune sera) and after three inoculations with the recombinant protein (hyperimmune sera).

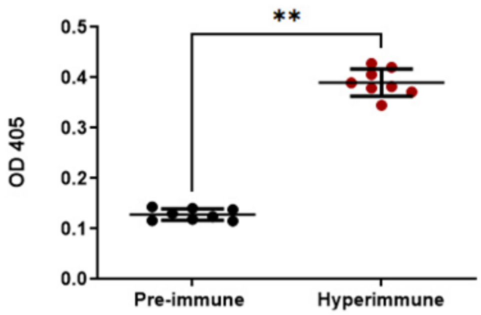

Supplementary Figure S1. IgG antibodies against the ORF8 protein. Preimmune vs. hyperimmune sera are shown. All data are mean ± SEM; n = 8. ** $p < 0.05$ paired t test.
